# Proton Beam Therapy for Head and Neck Carcinoma of Unknown Primary: Toxicity and Quality of Life

**DOI:** 10.14338/IJPT-20-00034.1

**Published:** 2021-06-25

**Authors:** Alexander D. Sherry, Dario Pasalic, G. Brandon Gunn, C. David Fuller, Jack Phan, David I. Rosenthal, William H. Morrison, Erich M. Sturgis, Neil D. Gross, Maura L. Gillison, Renata Ferrarotto, Adel K. El-Naggar, Adam S. Garden, Steven J. Frank

**Affiliations:** 1 Vanderbilt University School of Medicine, Nashville, TN, USA; 2 Department of Radiation Oncology, The University of Texas MD Anderson Cancer Center, Houston, TX, USA; 3 Department of Head and Neck Surgery, The University of Texas MD Anderson Cancer Center, Houston, TX, USA; 4 Department of Thoracic/Head and Neck Medical Oncology, The University of Texas MD Anderson Cancer Center, Houston, TX, USA; 5 Department of Pathology, The University of Texas MD Anderson Cancer Center, Houston, TX, USA

**Keywords:** patient-reported outcomes, head and neck cancer, intensity-modulated proton radiation therapy, sequelae

## Abstract

**Purpose:**

Proton radiation therapy (PRT) may offer dosimetric and clinical benefit in the treatment of head and neck carcinoma of unknown primary (HNCUP). We sought to describe toxicity and quality of life (QOL) in patients with HNCUP treated with PRT.

**Patients and Methods:**

Toxicity and QOL were prospectively tracked in patients with HNCUP from 2011 to 2019 after institutional review board approval. Patients received PRT to the mucosa of the nasopharynx, oropharynx, and bilateral cervical lymph nodes with sparing of the larynx and hypopharynx. Patient-reported outcomes were tracked with the MD Anderson Symptom Inventory–Head and Neck Module, the Functional Assessment of Cancer Therapy–Head and Neck, the MD Anderson Dysphagia Inventory, and the Xerostomia-Related QOL Scale. Primary study endpoints were the incidence of grade ≥ 3 (G3) toxicity and QOL patterns.

**Results:**

Fourteen patients (median follow-up, 2 years) were evaluated. Most patients presented with human papillomavirus–positive disease (n = 12, 86%). Rates of G3 oral mucositis, xerostomia, and dermatitis were 7% (n = 1), 21% (n = 3), and 36% (n = 5), respectively. None required a gastrostomy. During PRT, QOL was reduced relative to baseline and recovered shortly after PRT. At 2 years after PRT, the local regional control, disease-free survival, and overall survival were 100% (among 7 patients at risk), 79% (among 6 patients at risk), and 90% (among 7 patients at risk), respectively.

**Conclusion:**

Therefore, PRT for HNCUP was associated with highly favorable dosimetric and clinical outcomes, including minimal oral mucositis, xerostomia, and dysphagia. Toxicity and QOL may be superior with PRT compared with conventional radiation therapy and PRT maintains equivalent oncologic control. Further prospective studies are needed to evaluate late effects and cost-effectiveness.

## Introduction

Head and neck carcinoma of unknown primary (HNCUP) is a rare presentation [1] and high-level evidence from randomized trials for management is, therefore, lacking [2]. Management options include surgery alone, surgery followed by adjuvant radiation, radiation therapy alone, or radiation therapy plus systemic therapy. Because most HNCUPs are squamous cell carcinomas (SCCs), radiation has been the cornerstone of treatment since the 1970s, when patients were successfully treated with comprehensive radiation to the bilateral cervical lymph nodes, the entire pharyngeal axis, and the larynx [3–6]. However, even three-dimensional conformal radiation therapy led to an appreciable incidence of chronic xerostomia, dysphagia, skin desquamation, odynophagia, and percutaneous endoscopic gastrostomy (PEG) tube dependence [7].

Since then, several advances have led to improvements in the side-effect profiles and quality of life (QOL). Intensity-modulated radiation therapy (IMRT), in particular, offered superior organ sparing, resulting in reduced toxicity and improved patient-reported QOL [8, 9]. Developments in detecting occult primary disease through positron emission tomography (PET) and diagnostic laryngoscopy with biopsies or transoral robotic surgery, as well as recognition of human papillomavirus (HPV) status, have driven strategic reductions in radiation dose and volume [10–12]. As such, management goals have evolved toward preserving QOL and maintaining oncologic efficacy. In recognition of these factors, Kamal et al [13] recently reported excellent long-term oncologic control with larynx- and hypopharynx-sparing IMRT.

Further cumulative QOL improvements may be achievable with proton radiation therapy (PRT) [14]. Compared with photon-based radiation therapy, PRT affords superior conformality, with a steep lateral penumbra and lack of exit dose, which may obviate toxicity associated with nontarget beam paths in IMRT [15]. Toxicity may be further diminished with intensity-modulated PRT (IMPT), also known as active scanning or pencil-beam PRT [16,17]. In contrast to passive scatter PRT, IMPT allows precise dose painting of treatment volumes to help avoid healthy tissues by using beam steering [18, 19].

There is ample reason to suspect that treatment sequelae and QOL for patients with HNCUP can be further optimized with PRT [20–22]. Here, we report our initial outcomes, physician-assessed toxic effects, and patient-reported QOL after PRT for the treatment of HNCUP.

## Patients and Methods

### Patient Population

After institutional review board approval, we conducted a prospective observational study of adult patients with HNCUP who had received PRT at our high-volume tertiary cancer center from 2011 through 2019. Participants were enrolled on 1 of 2 protocols (NCT00991094: “Data Collection of Normal Tissue Toxicity for Proton Therapy” for 5 patients; and NCT01627093: “Medical Data Collection of Patients With Head and Neck Cancer Treated With Proton Therapy” for 9 patients). All patients gave signed, informed consent for the study. Patients with non-SCC histology were excluded; in addition, patients with mucosa-sparing treatment solely to the ipsilateral neck were excluded because those patients are not representative of conventional HNCUP.

Physician-assessed toxicity data were prospectively collected at baseline, weekly during PRT, and at follow-up visits according to the National Cancer Institute's Common Terminology Criteria for Adverse Events, version 4.0. Patient-reported outcomes (PROs) were prospectively measured at baseline and weekly during PRT by several validated methods. The MD Anderson Symptom Inventory–Head and Neck Module (MDASI-HN) and the Functional Assessment of Cancer Therapy–Head and Neck Cancer (FACT-H&N) evaluated general symptoms, psychosocial properties, pain, and function [23, 24]. The top 5 most-severe symptoms on MDASI-HN were averaged to generate a composite severe-symptom burden score, and the top 11 most-severe symptoms were averaged to create a composite moderate/severe score [25]. Data from the Xerostomia-related QOL scale (XeQoLS) and the MD Anderson Dysphagia Inventory (MDADI) were also collected [26, 27]. Based on validation studies, the minimum important difference (MID) in the MDADI 19-item composite score was defined as ≥ 10, and the MID for the FACT-H&N total score was also defined as ≥ 10 [28, 29]. FACT-H&N, XeQoLS, and MDADI surveys were also given during the subacute phase (during the first 3 months after the end of PRT) and the chronic period (from 3 months after PRT through last follow-up).

Disease was retrospectively staged according to the *AJCC Cancer Staging Manual*, 8th edition [30]. Dose-volume histograms were generated to evaluate mean dose (*D*_mean_) and maximum dose (*D*_max_) for organs at risk; the entire esophagus was contoured for dose-volume calculations.

### Treatment Techniques

Radiation therapy planning was done with a larynx-sparing and hypopharynx-sparing approach, as previously described [8, 13]. Our IMPT technique, used for all but 1 patient (93%) in this study, has also been previously reported [23, 31]. Briefly, all patients underwent a supine noncontrast computed tomography treatment simulation while immobilized with a thermoplastic mask and customized posterior head/neck/shoulder mold. A bite block, alone or with a tongue-depressing stent, was used for reproducibility and oral cavity sparing. The robustness of each treatment plan was assessed to evaluate its sensitivity to uncertainties associated with variations in patient setup and proton beam range. A relative biological effectiveness (RBE) value of 1.1 was used. The dose prescribed to the high-risk clinical target volume (CTV1), which included gross lymph node disease with a margin or the preoperative tumor bed with a margin, was 58-70 GyRBE; that to the intermediate-risk clinical target volume (CTV2), which included any neck volume at high risk for microscopic disease without clinical, radiologic, or pathologic evidence of disease, was 55-66 GyRBE; and that to the low-risk clinical target volume (CTV3), which included neck volume and mucosa considered to be at low risk of subclinical disease, was 50-56 GyRBE. Patients were treated with a simultaneous integrated boost to achieve the CTV1, CTV2, and CTV3 dose prescriptions. All patients were physically examined during a head and neck radiation oncology treatment planning and development clinic session, and the target volumes were assessed by ≥ 2 radiation oncologists for quality assurance [32]. The quantitative analysis of normal tissue effects (QUANTEC) was used as a guideline for organ-at-risk constraints, and dose to organs at risk was kept as low as reasonably achievable per the prescribing radiation oncologist. Kilovoltage image guidance was used before daily treatment. The addition of chemotherapy was individualized and based upon multidisciplinary consensus; typically, patients with high nodal disease burden, indicated by multiple bulky lymph nodes, extracapsular extension, and/or N3 disease, were treated with induction or concurrent systemic therapy. After nodal dissection, concurrent chemotherapy with radiation was considered for extensive extracapsular extension.

### Study Endpoints

Primary endpoints were incidence of acute G3 toxicity and QOL patterns. Secondary endpoints included dose distribution and rates of local-regional control (LRC), disease-free survival (DFS), and overall survival (OS). The LRC was defined as the absence of radiologic or pathologic evidence of disease in the head and neck. Time to DFS was defined as the time from end of PRT until clinically or radiologically suspected local-regional or distant failure or death. The OS was defined as the time from end of PRT to death.

### Statistical Analysis

Kaplan-Meier analysis was used to calculate LRC, DFS, and OS. Symptom burden for all PROs was compared over time with the Mann-Whitney test for 2 comparisons or the Kruskal-Wallis test for > 2 comparisons. Binary logistic regression was used to evaluate the relationship between clinical variables and incidence of G3 toxicity. To characterize the evolution and resolution of toxic effects, a Markovian nonparametric multistate survival analysis was constructed with the msSurv R package [33]. The state occupational probability of having a toxic effect over time by grade was calculated by the Aalen-Johansen estimator [34]. All tests were two-sided with an α level of 0.05, and all confidence intervals were reported at 95% (95% CI). Analyses and plots were completed with SAS version 9.4 (SAS Institute, Cary, NC), R version 3.4 (R Foundation for Statistical Computing, Vienna, Austria), and GraphPad Prism 8 (GraphPad Software, La Jolla, California) software.

## Results

### Patient Clinicopathologic Details

Twenty-three patients were assessed for study eligibility. After exclusion of 2 patients (9%) who did not receive PRT and 7 patients (30%) treated to the ipsilateral neck only, the 14 patients analyzed were 12 men (86%) and 2 women (14%) (Table 1). Median age at the time of PRT was 62 years (range, 26-72 years). Most patients had HPV-positive SCC (n = 12, 86%), and most patients were diagnosed with cN1 disease (n = 12, 86%). Workup for all patients included PET/computed tomography (CT) scan and exam under anesthesia with direct laryngoscopy and biopsies. Before PRT, 2 patients (14%) underwent neck dissection: the first patient had 3/34 positive nodes without extracapsular extension removed, and the second patient had 2/33 positive nodes with extracapsular extension removed. Seven patients (50%) underwent tonsillectomy, which was negative for primary disease in all cases. Four patients (29%) received induction systemic therapy for multiple bulky lymph nodes (n = 4), and three patients (21%) had concurrent chemotherapy with PRT for extensive postsurgical extracapsular extension (n = 1) or multiple bulky lymph nodes (n = 2).

**Table 1.** Patient clinicopathologic characteristics.

**Characteristic**	**Patients, No. (%), n = 14**
Age, median [range], y	62 [26-72]
Sex	
Men	12 (86)
Women	2 (14)
Race	
White	13 (93)
Black	1 (7)
Smoking status	
Current	0 (0)
Former	8 (57)
Never	6 (43)
Alcohol consumption	
Current	10 (71)
Former	1 (7)
Never	3 (21)
Method of diagnosis	
Fine-needle aspiration	11 (79)
Excisional biopsy	3 (21)
HPV status	
HPV^+^ SCC	12 (86)
HPV unknown SCC	2 (14)
Tonsillectomy	
Yes	7 (50)
Ipsilateral, n = 7	2 (29)
Bilateral, n = 7	5 (71)
No	7 (50)
Clinical stage (AJCC 8th)	
I	11 (79)
II	1 (7)
III	1 (7)
IVA	1 (7)
Clinical lymph node status (AJCC 8th)	
cN1	12 (86)
cN2	2 (14)
Size of largest lymph node, median [range], cm	3.1 [2.0-5.2]
No. of involved neck levels	
1	9 (64)
≥ 2	5 (36)
Pre-RT neck dissection	
Selective	2 (14)
No	12 (86)
Technique	
Passive scatter	1 (7)
IMPT	13 (93)
Intraoral stabilization	
Bite block	10 (71)
Bite block and stent	4 (29)
Overall RT time, median [range], days	42 [38-50]
Induction systemic therapy	
Yes	4 (29)
No	10 (71)
Type of induction systemic therapy, n = 4	
Platinum + taxane	2 (50)
Platinum + taxane + fluorouracil	2 (50)
Concurrent chemotherapy	
Yes	3 (21)
No	11 (79)
Type of concurrent chemotherapy, n = 3	
Platinum	2 (66)
Cetuximab	1 (33)

**Abbreviations:** HPV, human papillomavirus; SCC, squamous cell carcinoma; RT, radiation therapy; IMPT, intensity-modulated proton therapy**.**

### Proton Radiation Therapy

Most patients (n = 13, 93%) were treated with IMPT; 1 patient (7%), the earliest in the cohort, received passive scatter PRT. Median doses prescribed to the CTV1, CTV2, and CTV3 were 66 GyRBE (range, 58-70 GyRBE), 60 GyRBE (range, 55-66 GyRBE), and 54 GyRBE (range, 50-56 GyRBE); 1 patient (7%) with cN2a disease was prescribed 58 GyRBE to the CTV1 after an R0 resection without adverse pathologic features. One patient (7%) required a 3-day break during treatment for psychiatric reasons, but that patient received all prescribed fractions.

**Figure 1.**
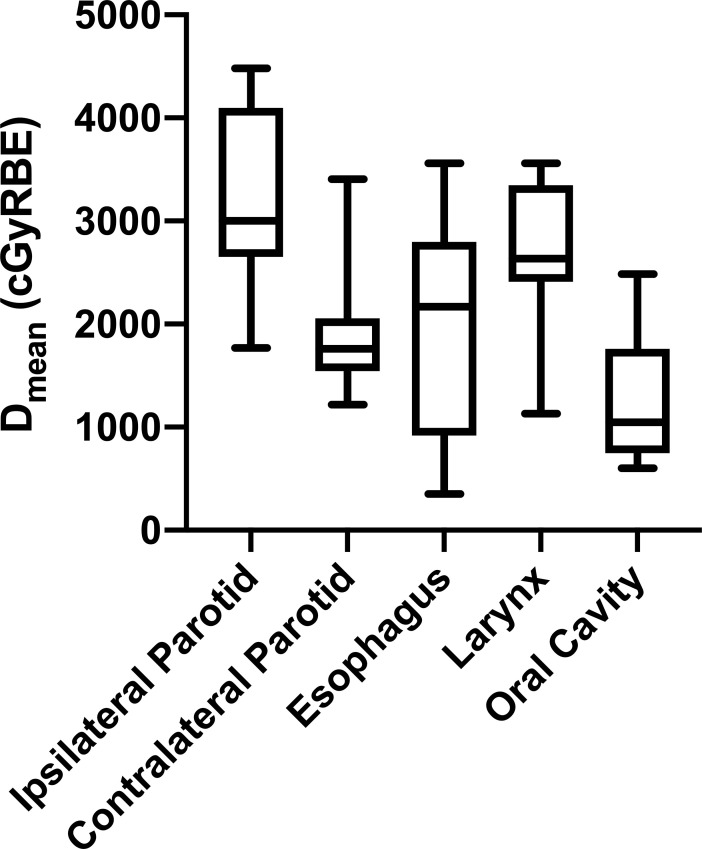
Mean dose (D_mean_) in cGyRBE for organs at risk obtained by dose-volume histogram analysis and displayed as interleaved box and whiskers plots. Error bars represent the minimum and maximum D_mean_ observed for each organ at risk, and the interquartile range of D_mean_ is shown by the extent of each box, with the median represented by the solid line within each box.

*D*_mean_ for the parotid, esophagus, oral cavity, and larynx are shown in Figure 1. The mean ipsilateral parotid *D*_mean_ was 3228 cGyRBE (range, 1770-4482 cGyRBE), and the mean *D*_mean_ to the contralateral parotid was 1869 cGyRBE (range, 1219-3407 cGyRBE). Only 1 patient (7%) had a parotid *D*_mean_ that exceeded 25 GyRBE bilaterally; that patient had presented with bilateral cervical nodal disease. Mean larynx *D*_mean_ was 2684 cGyRBE (range, 1133-3560 cGyRBE), mean esophagus *D*_mean_ was 1946 cGyRBE (range, 353-3561 cGyRBE), and mean oral cavity *D*_mean_ was 1238 cGyRBE (range, 604-2486 cGyRBE).

### Clinician-Assessed Toxic Effects

**Figure 2.**
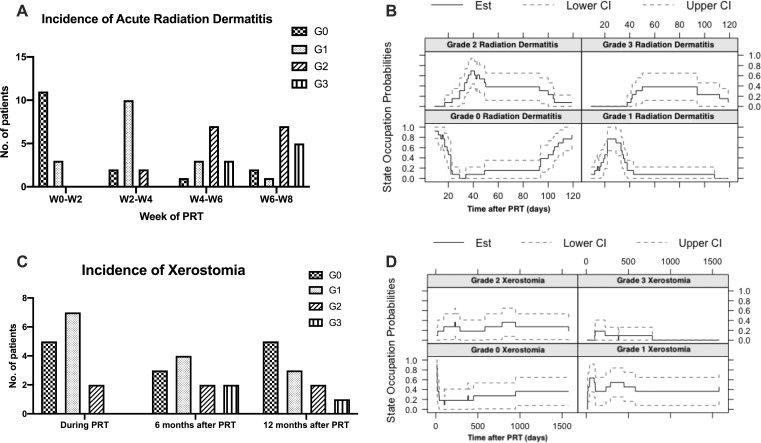
Clinician-derived assessments of toxicity as a function of time during proton radiation therapy (PRT) graded according to the Common Terminology Criteria for Adverse Events version 4.0 for (A) radiation dermatitis and (C) xerostomia. Each patient is represented by the highest grade observed during the period marked on the x-axis. The stated occupational probabilities over time per grade for (B) radiation dermatitis and (D) xerostomia were estimated by using the Aalen-Johansen estimator and shown with 95% confidence intervals.

At the per-patient level, the highest-grade toxicity experienced was G3 for 8 patients (57%), G2 for 5 patients (36%), and G1 for 1 patient (7%). Patients treated with chemotherapy were less likely to experience G3 toxicity (13% versus 88%, unadjusted odds ratio [OR] 0.04, 95% CI 0.002-0.74, *P* = .03). The most common side effects were radiation dermatitis (93%; n = 13), oral mucositis (93%; n = 13), and dry mouth (79%; n = 11). Radiation dermatitis was the most common G3 toxicity, noted in 36% of patients (n = 5; Table 2). Both the incidence and severity of radiation dermatitis increased over time during PRT (**Figure 2A**). The estimated probabilities of G3 radiation dermatitis at 3 weeks, 5 weeks, and 7 weeks after the start of PRT were 0% (95% CI, 0%-0%), 0% (95% CI, 0%-0%), and 38.5% (95% CI, 12%-65%) (**Figure 2B**). Experiencing G3 radiation dermatitis was not associated with either dose to a skin volume of 0.5 cm^3^ (unadjusted OR, 1.00; 95% CI, 0.997-1.002; *P* = .75) or the skin *D*_max_ (unadjusted OR, 1.00; 95% CI, 0.998-1.005; *P* = .42).

**Table 2.** Physician-assessed toxicity assessed by the National Cancer Institute's Common Terminology Criteria for Adverse Events version 4.0; n = 14.

**Toxicity**	**Grade 0, No. (%)**	**Grade 1, No. (%)**	**Grade 2, No. (%)**	**Grade 3, No. (%)**	**Grade 4 or 5, No. (%)**
Constitutional and general disorders					
Fatigue	5 (36)	8 (57)	1 (7)	0 (0)	0 (0)
Weight loss	7 (50)	2 (14)	4 (29)	1 (7)	0 (0)
Pain	5 (36)	1 (7)	5 (36)	3 (21)	0 (0)
Nervous system disorders					
Headache	9 (64)	5 (36)	0 (0)	0 (0)	0 (0)
Dysgeusia	1 (7)	2 (14)	11 (79)	0 (0)	0 (0)
Peripheral motor and sensory (cranial) neuropathy	14 (100)	0 (0)	0 (0)	0 (0)	0 (0)
Gastrointestinal disorders					
Nausea	6 (43)	8 (57)	0 (0)	0 (0)	0 (0)
Vomiting	13 (93)	1 (7)	0 (0)	0 (0)	0 (0)
Dysphagia	5 (36)	5 (36)	4 (29)	0 (0)	0 (0)
Mucositis oral	1 (7)	5 (36)	7 (50)	1 (7)	0 (0)
Dry mouth	3 (21)	5 (36)	3 (21)	3 (21)	0 (0)
Constipation	12 (86)	2 (14)	0 (0)	0 (0)	0 (0)
Metabolism and nutrition disorders					
Dehydration	11 (79)	3 (21)	1 (7)	0 (0)	0 (0)
Injury, poisoning, and procedural complications					
Dermatitis, radiation-related	1 (7)	2 (14)	6 (43)	5 (36)	0 (0)
Infections and infestations					
Infection	14 (100)	0 (0)	0 (0)	0 (0)	0 (0)
Respiratory, thoracic, and mediastinal disorders					
Stridor	14 (100)	0 (0)	0 (0)	0 (0)	0 (0)
Dyspnea	14 (100)	0 (0)	0 (0)	0 (0)	0 (0)

During the entire follow-up period, the incidence of G3 xerostomia was 21% (n = 3). The crude proportions of patients with G3 xerostomia during PRT, 6 months after treatment, and 1 year after treatment were 0% (0 of 14), 18% (2 of 11), and 9% (1 of 11) (**Figure 2C).** The occupational probabilities of G3 xerostomia at 5 weeks, 4 months, and 1 year after PRT, calculated with 95% CIs with an Aalen-Johnson estimator were 0% (95% CI, 0%-0%), 18% (95% CI, 0%-41%), and 9% (95% CI, 0%-26%) (**Figure 2D**). The G3 oral mucositis was observed in 1 patient (7%). No instances of cranial neuropathy, stridor, dyspnea, or hospitalization were observed. No patients had a PEG tube placed before, during, or after PRT.

### Patient-Reported Outcomes

Regarding PRO response rate, the MDASI-HN was completed by 7 patients (50%) at baseline and by 8 patients (57%) weekly during PRT. The XeQoLS was completed at baseline by 7 patients (50%), weekly during treatment by 7 patients (50%), during the subacute phase by 5 patients (36%), and during the chronic phase by 4 patients (29%). The MDADI was completed by 7 patients (50%) at baseline, 7 patients (50%) weekly on treatment, 5 patients (36%) in the subacute phase, and 4 patients (29%) in the chronic phase. The FACT-H&N was completed by 6 patients (43%) at baseline, 7 patients (50%) weekly on treatment, 5 patients (36%) in the subacute phase, and 4 patients (29%) in the chronic phase.

**Figure 3.**
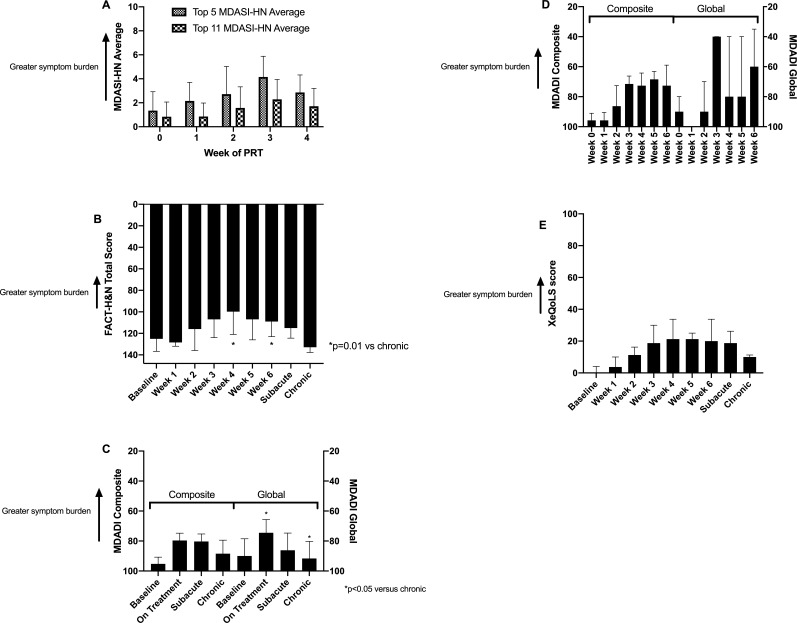
Patient-reported outcomes before, during, and after proton radiation therapy (PRT) suggest excellent quality of life (QOL) and low symptom burden. (A) Overall QOL specific to patients with head and neck cancer as shown by the MD Anderson Symptom Inventory (MDASI), quantified by the top 5 most-prominent symptoms (moderate/severe symptom burden) and the top 11 most-prominent symptoms (overall symptom burden) through week 4 of PRT, with the lower scores representing higher QOL on a scale of 0 to 10. Data are shown as means with error bars representing 95% confidence intervals. (B) Overall QOL specific to patients with head and neck cancer as shown by the Functional Assessment of Cancer Therapy–Head and Neck Cancer (FACT-HN), with higher scores representing higher QOL on a scale of 0 to 144. Median and interquartile range are shown. P values were derived from Mann-Whitney tests. (C) MD Anderson Dysphagia Inventory (MDADI) composite and global scores, shown as median and interquartile range, during all study phases and (D) during treatment on a scale of 0 to 100, with higher scores representing higher QOL. P values were derived from Mann-Whitney tests. (E) Xerostomia as reported by the Xerostomia-related quality of life scale (XeQoLS) is shown as median and interquartile range on a scale of 0 to 100, with lower scores representing lower symptom burden. P values were derived from Mann-Whitney tests.

The top-5 MDASI-HN score peaked numerically during week 3 of PRT, although the MDASI-HN score did not differ significantly between weeks of PRT (*P* = .11 for the top 5 MDASI-HN, *P* = .43 for the top 11 MDASI) (**Figure 3A**). The highest mean score was 4/10 for the top 5 MDASI-HN and 2/10 for the top 11 MDASI-HN in week 3 of PRT, indicating a low burden of severe or moderate/severe symptoms. The FACT-HN showed statistical differences between time points (*P* = .03) as well as statistical differences between week 4 (*P* = .01) and week 6 (*P* = .01) compared with the chronic period (**Figure 3B**). The FACT-HN scores also exceeded the MID, with a median score of 125/144 (95% CI, 99-139) before PRT and nadir median score of 100/144 (95% CI, 80-126) in week 6 of PRT, suggesting that statistically significant score changes were also clinically meaningful. However, the FACT-HN score seemed to recover during the subacute phase (median, 115/144; 95% CI, 95-126) to match the baseline QOL. The QOL even exceeded the MID during the chronic phase (median, 133/144; 95% CI, 119-138) compared with baseline, suggesting a clinically meaningful improvement in QOL starting 3 months after PRT compared with before treatment.

The MDADI composite score decreased beyond the MID during PRT (median, 80/100; 95% CI, 71-92), remained stable during the subacute period (median, 80/100; 95% CI, 75-87), and recovered to baseline levels (median, 95/100; 95% CI, 89-100) during the chronic period (median, 88/100; 95% CI, 81-98) (**Figure 3C**). The MDADI global score was not significantly different comparing all phases (*P* = .14) but was lower during treatment (median score of 75/100; 95% CI, 60-100) than during the chronic period (median score of 92/100; 95% CI, 80-100) (*P* = .04) (**Figure 3C**). During treatment, the MDADI composite score decreased below the MID, beginning in week 3 (median score. 71/100; 95% CI, 64-96), and this pattern was statistically different comparing all weeks (*P* = .02) (**Figure 3D**). Weekly global score patterns followed the composite score patterns, with clinically significant score decrements in weeks 3 to 6 compared with baseline (**Figure 3D**).

The XeQoLS score increased from baseline (median, 0/100; 95% CI, 0-13) progressively with each week of treatment (*P* = .0002 between time points), peaking in week 4 with a median 21/100 score (95% CI, 13-36), followed by an apparent recovery during the chronic period (median, 10/100; 95% CI, 4-31) (**Figure 3E**).

### Disease Control and Survival

**Figure 4.**
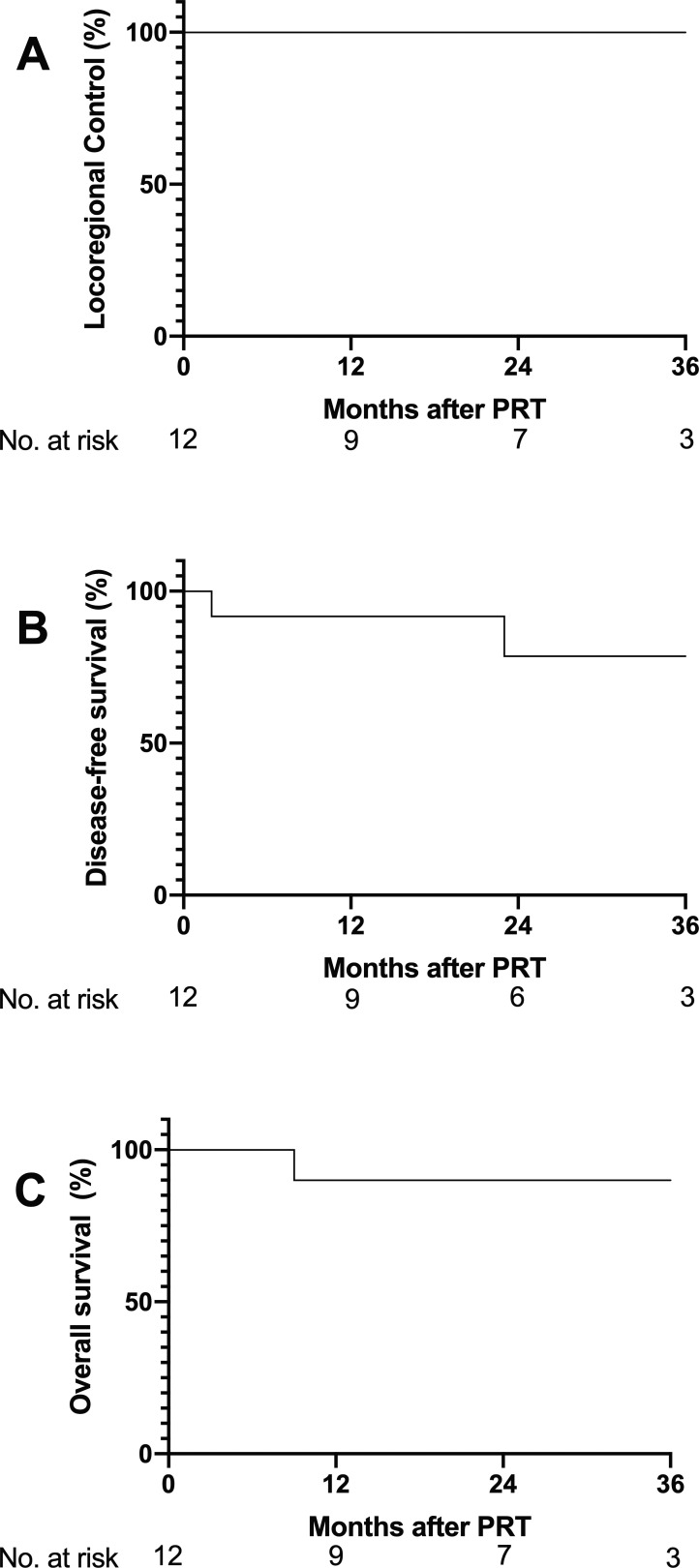
Kaplan-Meier plots displaying (A) local regional control, (B) disease-free survival, and (C) overall survival from the end of proton radiation therapy (PRT). Numbers of subjects at risk are indicated under the x-axis.

Two patients (14%) were excluded from survival analysis because of lack of follow-up. The median follow-up time for the other 12 patients (86%) was 27 months (range, 2-86 months). The LRC rates were 100% (9 at risk) at 1 year (95% CI, 100%-100%), 100% (7 at risk) at 2 years (95% CI, 100%-100%), and 100% (3 at risk) at 3 years (95% CI, 100%-100%) (**Figure 4A**). No in-field or out-of-field local regional failures occurred. Two patients (17%) experienced distant failures, one who received induction chemotherapy, followed by chemoradiation for HPV-positive SCC; and one who received chemoradiation for HPV-positive SCC. The DFS rates were 92% (9 at risk) at 1 year (95% CI, 54%-97%), 79% (6 at risk) at 2 years (95% CI, 35%-94%), and 79% (3 at risk) at 3 years (95% CI, 35%-94%) (**Figure 4B**). The OS rates were 90% (9 at risk) at 1 year (95% CI, 47%-98%), 90% (7 at risk) at 2 years (95% CI, 47%-98%), and 90% (3 at risk) at 3 years (95% CI, 47%-98%) (**Figure 4C**).

## Discussion

Our findings suggest that PRT for HNCUP delivers low doses to healthy tissues with correspondingly low toxicity, excellent patient-reported QOL, and retained oncologic efficacy. Although further studies are needed to validate the hypothesis that PRT, particularly IMPT, improves sequelae and QOL relative to IMRT, PRT is entirely compatible with other modern toxicity-reducing techniques, including dose and volume reduction, careful patient selection, and complete diagnostic workup. The PRT seems to be the next natural step in furnishing a patient-centered and toxicity-reducing treatment for HNCUP [35].

Although the limited number of prospective HNCUP studies makes comparison of our toxicity findings with other approaches challenging, PRT seems to substantially reduce G3 oral mucositis compared with IMRT. A prospective evaluation of IMRT reported an acute G3 oral mucositis rate of 52% [36]. In contrast, we observed a rate of G3 oral mucositis of 7% (1 of 14 patients), which could be attributed to the ability of PRT to spare the oral cavity even when the oropharynx is targeted. Remarkably, we also found an absence of G3 dysphagia and PEG placement, which compares favorably with a study of IMRT in which 40% of patients required PEG placement [13]. Our PRO results corroborate this finding because the MDADI demonstrated transient, mild dysphagia that returned to baseline within weeks of completing PRT, likely reflecting esophageal sparing. This lack of acute, as well as chronic, dysphagia (experienced by up to 7% [n = 1] of the patients treated with IMRT) allows patients to continually exercise the pharyngeal neuromusculature, promoting swallowing and long-term functionality [13]. Studies of other head and neck malignancies are consistent with this finding. For example, Holliday et al [37] found that IMPT reduced the incidence of PEG tubes, with lower oral cavity and mandibular mean doses in nasopharyngeal cancer relative to IMRT. Similarly, Blanchard et al [38] reported that, in oropharyngeal cancer, IMPT led to lower rates of PEG tube placement and severe weight loss compared with IMRT.

Only 18% of patients in our study (2 of 11) had G3 xerostomia at 6 months after PRT, a rate that seems promising compared with 27% to 36% of patients with G3 xerostomia at 6 months after IMRT [36, 39]. Our PRO results also indicated a low burden of xerostomia and resolution of XeQoLS scores to baseline. This benefit is probably a consequence of dose reduction to the contralateral parotid gland and minor salivary glands in the oral cavity. The *D*_mean_ for 93% of our patients (all except for 1 [7%] with bilateral cervical nodal disease) satisfied the QUANTEC criterion of *D*_mean_ < 25 Gy for both parotid glands, which compares favorably to other studies of IMRT in which this criterion was satisfied in just 32% of patients [40, 41]. Across all domains, PRO findings indicated excellent QOL before, during, and after PRT. The MDASI-HN and FACT-HN results suggested only a mild decrement during treatment.

Although rates of G3 oral mucositis, dysphagia, and xerostomia seem to be better with PRT than with IMRT, rates of G3 dermatitis with PRT may be higher than those with IMRT. In one study of IMRT, 28% of patients had acute G3 dermatitis versus our finding of 36% (5 of 14 patients) [36]. Passive scatter PRT for superficial targets, such as cervical lymph nodes, has been linked with dermatitis that is comparable to or worse than that occurring after photon radiation therapy [42]. However, all but 1 patient in our study (93%; 13 of 14) was treated with IMPT, which has been associated with superior skin sparing compared with passive scatter. Nevertheless, until a more robust evaluation is completed in an ongoing phase II/III study (NCT01893307), patients should be counseled regarding the potential tradeoff between acute dermatologic toxicity and improvements in oral mucositis, xerostomia, and dysphagia [43]. Measures for reducing dermatitis from PRT warrant continued investigation [44].

We are the first to report, to our knowledge, that PRT is associated with excellent oncologic outcomes in HNCUP, although our findings are limited by small sample size and short follow-up. At 2 years, LRC was 100% (with 7 patients at risk), DFS was 79% (6 at risk), and OS rate was 90% (7 at risk). Our lack of local-regional failures suggests that patients treated with this larynx- and hypopharynx-sparing PRT approach do not seem to be at increased risk of failure relative to other techniques. Preclinical data suggest that PRT may be more effective at inducing mitotic catastrophe and tumor senescence in head and neck SCC than photon radiation therapy, although this hypothesis has not been validated clinically [45]. A secondary endpoint of an ongoing phase II/III study is attempting to address that question [43].

Other limitations to this observational study include the study design. Although toxic effects and PROs were prospectively collected, treatments were nonblinded and nonrandomized, increasing the risk of bias. Poor compliance with PRO completion in our study may have biased the PRO benefits reported here. Furthermore, given the rarity of prospective HNCUP studies and the even greater rarity of PRO assessments, comparisons with normative data are challenging, although QOL is critical to report and seems robust based on validated score interpretations for other head and neck diseases [46, 47]. Our sample size limited power to analyze predictors of toxicity and precluded multivariable analysis to evaluate for confounding effects, limiting the conclusions of the hypothesis testing. Follow-up was also heterogeneous; we will continue long-term investigation of these patients to evaluate late effects because > 28% of patients treated with IMRT may experience chronically impaired function and QOL [48, 49]. Given the generally long-term survival after HNCUP, PRT may offer an even greater clinical benefit than is shown here by mitigating late toxicity and improving cost-effectiveness, although this requires further study [13, 15]. Finally, because most patients in this study had HPV-positive disease without a smoking history, the results of this study may not be extrapolatable to patients with HPV-negative disease with a smoking history.

In summary, we report the first prospective study of PRT for the treatment of HNCUP. Our findings suggest that PRT offers a highly conformal method of treating large, complex target volumes with excellent LRC and sparing of normal structures. Moreover, PRT holds considerable promise for maximizing QOL and minimizing sequelae, including dysphagia, oral mucositis, PEG placement, and xerostomia. We will continue to collect prospective data on this evolving cohort to investigate long-term toxicity, effects on QOL, and cost-effectiveness.
